# Dispositional optimism and depression risk in older women in the Nurses´ Health Study: a prospective cohort study

**DOI:** 10.1007/s10654-021-00837-2

**Published:** 2022-01-15

**Authors:** Jakob Weitzer, Claudia Trudel-Fitzgerald, Olivia I. Okereke, Ichiro Kawachi, Eva Schernhammer

**Affiliations:** 1 Department of Epidemiology, Center for Public Health, Medical University of Vienna, Vienna, Austria; 2 Department of Social and Behavioral Sciences, Harvard T.H. Chan School of Public Health, Boston, USA; 3 Lee Kum Sheung Center for Health and Happiness, Harvard T.H. Chan School of Public Health, Boston, USA; 4 Department of Psychology, Université du Québec à Trois-Rivières, Québec, Canada; 5 Research Center of Institut Universitaire en Santé Mentale de Montréal, Québec, Canada; 6 Massachusetts General Hospital and Harvard Medical School, Boston, MA USA; 7 Department of Epidemiology, Harvard T.H. Chan School of Public Health, Boston, MA USA; 8 Channing Division of Network Medicine, Department of Medicine, Brigham and Women’s Hospital and Harvard Medical School, 181 Longwood Ave, Boston, MA 02115 USA

**Keywords:** Optimism, Depression, NHS1, Positive psychology, Best possible self

## Abstract

Dispositional optimism is a potentially modifiable factor and has been associated with multiple physical health outcomes, but its relationship with depression, especially later in life, remains unclear. In the Nurses´ Health Study (n = 33,483), we examined associations between dispositional optimism and depression risk in women aged 57–85 (*mean* = 69.9, *SD* = 6.8), with 4,051 cases of incident depression and 10 years of follow-up (2004–2014). We defined depression as either having a physician/clinician-diagnosed depression, or regularly using antidepressants, or the presence of severe depressive symptoms using validated self-reported scales. Age- and multivariable-adjusted Cox proportional hazards models were used to estimate hazard ratios (HRs) with 95% confidence intervals (95% CIs) across optimism quartiles and for a 1-standard deviation (*SD*) increment of the optimism score. In sensitivity analyses we explored more restrictive definitions of depression, potential mediators, and moderators. In multivariable-adjusted models, women with greater optimism (top vs. bottom quartile) had a 27% (95%CI = 19–34%) lower risk of depression. Every 1-SD increase in the optimism score was associated with a 15% (95%CI = 12–18%) lower depression risk. When applying a more restrictive definition for clinical depression, the association was considerably attenuated (every 1-SD increase in the optimism score was associated with a 6% (95%CI = 2–10%-) lower depression risk. Stratified analyses by baseline depressive symptoms, age, race, and birth region revealed comparable estimates, while mediators (emotional support, social network size, healthy lifestyle), when combined, explained approximately 10% of the optimism-depression association. As social and behavioral factors only explained a small proportion of the association, future research should investigate other potential pathways, such as coping strategies, that may relate optimism to depression risk.

**Supplementary Information:**

The online version contains supplementary material available at 10.1007/s10654-021-00837-2 .

## Introduction

One in five individuals experience at least one major depressive episode in their lifetime. Of these, 80% have another episode, and 25% develop chronic symptoms (≥ 2 years) [1], often remaining undetected and untreated [2]. Because of its high societal burden, lifelong nature, difficulties in detection and treatment, and association with adverse health outcomes (e.g., cardiovascular disease [3, 4]), prevention should be given top priority [5].

Individuals with higher dispositional optimism—i.e., high expectations for positive outcomes in the future and low expectations for negative events [6]—experience significantly lower risk of depression and related outcomes [7–10], partially explained by better coping [11, 12], receiving more social support [13, 14] and a healthier lifestyle [15]. Optimism is strongly related to four of the Big Five factors of personality, with neuroticism and extraversion explaining by far the largest proportion of variance in optimism compared to agreeableness and conscientiousness [16]. Optimism can be enhanced via training [17]; thus, it is a modifiable factor that may help to prevent depression.

Despite a large body of literature on the association of dispositional optimism and depression-related outcomes, evidence from longitudinal studies with detailed confounder adjustment remains scarce. To date, four prospective studies have assessed the association between dispositional optimism and *incident* depression [7–10]. Three were based on the same adult population (mean age 44 years) of the Finnish Public Sector Study, showing high optimism to be associated with significantly lower risk of depressive disorder [8], work disability due to depression [9], and starting antidepressant medication treatment [7]. Among 464 elderly men (mean age 70.8 years), high vs. low optimism predicted a lower cumulative incidence of depressive symptoms over 15 years of follow-up, after detailed control for confounding [10]. To date, no study has examined this association among women of comparable age, who are generally more likely to experience depression [18, 19].

We examined in a large prospective cohort of middle-aged and older US women whether dispositional optimism predicted risk of incident depression in later life. We investigated effect modification by race, region of birth [20], age and baseline depressive symptoms and evaluated mediation by behavioral and social factors [21]. To minimize bias through the inclusion of potentially misclassified depression cases we conducted sensitivity analyses restricting the depression definition to physician diagnosed cases with anti-depressant use. Nonetheless, given the high validity of many of the outcomes including depression in the Nurses’ Health Study cohorts, we did not expect that applying a more stringent definition of depression would substantially alter our results.

## Methods

### Study population

The Nurses’ Health Study (NHS) began in 1976 when 121,700 U.S. female nurses aged 30–55 years returned a mailed questionnaire regarding socio-demographics, lifestyle, and medical history. Biennial follow-up questionnaires queried information on psychosocial factors, including depression and optimism, as well as incident medical conditions. Voluntary return of the questionnaires implies informed consent**;** the study protocol was approved by the institutional review boards of the Brigham and Women’s Hospital and Harvard T.H. Chan School of Public Health, and those of participating registries as required.

### Dispositional optimism

Dispositional optimism was assessed in 2004, 2008, and 2012 using the Life Orientation Test-Revised (LOT-R), the most commonly used, validated self-reported questionnaire to assess optimism. Six statements are rated with responses on a 5-point Likert Scale (0–4) rendering a scale from 0 to 24, with higher scores indicating higher optimism [22]. Quartiles were calculated to reduce the impact of influential outliers and to be able to distinguish effects, for example, between the least optimistic (Q1) vs. most optimistic (Q4) and least optimistic (Q1) vs. less optimistic (Q2) rather than merely looking at a one unit or a one *SD* increase in the optimism score. Overall stability across the three assessments of optimism was good (ICC = 0.59). Cronbach´s alpha for the assessment at baseline was 0.71.

### Depression

Depression was defined as clinician-diagnosed depression, regular antidepressant use, or the presence of severe depressive symptoms, as used in previous studies [18, 20, 21]. In sensitivity analyses, to minimize potential misclassification bias, two more restrictive definitions of depression were applied, 1. More restrictive: clinician-diagnosed depression or antidepressants use; 2. Most restrictive: clinician-diagnosed depression and antidepressants use [23].

Self-reported clinician diagnosis of depression was assessed biennially since 2000 and newly reported regular use of antidepressants was assessed biennially since 1996. Depressive symptoms were assessed in 1992, 1996 or 2000 using the 5-item Mental Health Index from the SF-36 scale (MHI-5; severe depressive symptoms: score ≤ 52) [24], in 2004 using the 10-item Center for Epidemiological Studies-Depression scale (CES-D-10; severe depressive symptoms: score ≥ 10) [25], and in 2008, 2012 and 2014 with the fifteen-item Geriatric Depression Scale (GDS-15; severe depressive symptoms: score ≥ 6) [26]. The comparability of these three measures in the NHS has been shown previously [20]. Selective serotonin reuptake inhibitors and other antidepressant classes except tricyclic antidepressants (TCAs) were considered as qualifying antidepressants, since we previously found that TCAs were more likely to be prescribed for indications other than depression [23].

### Covariables

Race, region of birth, highest education, husband´s highest education and father´s occupation were assessed in 1992. In 2000 the nurse´s subjective societal position [27], bodily pain and problems falling asleep or maintaining sleep were assessed, and in 2002 information on care for grandchildren, care for a disabled/ill person, and sleep duration was available. Work status, marital status, living arrangement, social-emotional support [28], the social network index [29], current physical functioning [30], comorbidity burden [21] and minor tranquilizer/benzodiazepine use were assessed at the 2004 analytic baseline and afterwards every 2–4 years. BMI, smoking status, and physical activity were assessed at baseline and thereafter every 2–4 years. Diet quality and alcohol consumption were assessed in 2002 and thereafter every 4 years. The selection of covariables was based on previous studies in the NHS [20, 21].

### Analytic sample

**Fig. 1:**
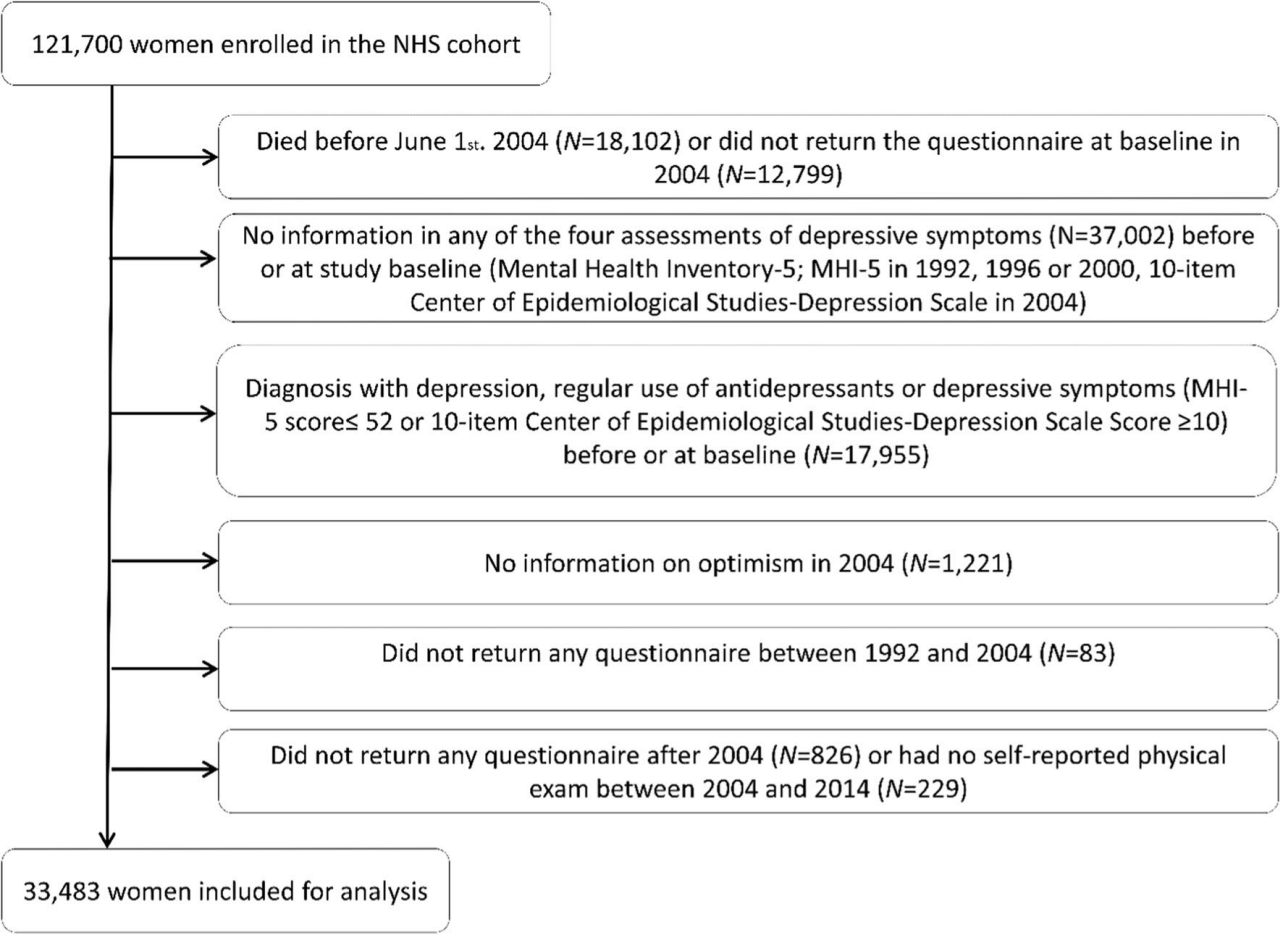
Study Flow Diagram illustrating the Nurses´ Health Study Cohort Exclusions at Study Baseline in 2004

Our base population comprised women who had returned the baseline questionnaire in 2004 (*N* = 90,799; Fig. 1), excluding participants with missing information on any depression items in 1992, 1996 or 2000 (*N* = 33,156), missing information on depressive symptoms at baseline (*N* = 3,846), who indicated a self-reported diagnosis of depression, a regular use of antidepressants, or severe depressive symptoms (MHI-5 score ≤ 52) prior to June 1, 2004 (*N* = 14,346), and participants with severe depressive symptoms at baseline (CESD-10 score ≥ 10; N = 3,609).

Further, we excluded participants (*N* = 1,221) with missing information on some or all optimism items in 2004, and participants (*N* = 1,138) who had not returned all questionnaires between 1992 and 2004, had not returned any questionnaire after 2004 or had no physical exam between 2004 and 2014, leaving a final analytic sample of 33,483 participants. Participants with missing information on optimism or missing information on depressive symptoms before or at baseline were slightly older, less educated and reported worse physical health compared to those included in the current analytic sample (sTable 1).

## Statistical analysis

We calculated age- and multivariable adjusted Cox Proportional hazard models to estimate hazard ratios (HRs) with 95% confidence intervals (95%CIs) across baseline optimism quartiles [Q_1(least optimistic)_ to Q_4(most optimistic)_] and for an increase of one standard deviation (SD) of the baseline optimism z-score in relation to depression incidence. We used age (in months) as the time scale in our models, and calculated person-time from the return date of the baseline questionnaire (2004) through the end of follow-up (June 1, 2014), date of depression diagnosis, death, or loss to follow-up, whichever occurred first. Multivariable models were adjusted for age, baseline depressive symptom score, educational status, birth region, race, subjective societal status/social standing, work status, living arrangement, marital status, husband´s education, father´s occupation, bodily pain, physical functioning, sleep duration, problems falling asleep or maintaining sleep, providing care for grandchildren or an ill/disabled person, multiple comorbidity and minor tranquilizer use [21]. Covariables were used at baseline and updated at each follow-up cycle; missing indicators were utilized to represent missing data in statistical models. In further analyses we estimated these associations with two more restrictive depression definitions: 1. More restrictive: clinician-diagnosed depression *or* antidepressants use; 2. Most restrictive: clinician-diagnosed depression *and* antidepressants use using separate models for each definition.

In secondary analyses, we estimated the proportion of the association between optimism and depression risk mediated by time-updated potential mediators (social-emotional support [13, 14, 28], social network index [28], and lifestyle [15]) using the publicly available %Mediate macro (https://www.hsph.harvard.edu/donna-spiegelman/software/mediate/) [31]. We stratified our models by baseline depressive symptoms [CESD-10 score: very low (< 3); low (3–5); moderate (6–10)], race [Non-Hispanic White; other], region of birth [West; Midwest; Northeast; South] [20] and age [< 65; 65–74; > 74 years] testing for heterogeneity using the likelihood ratio test. Because optimism may be higher among women without depression and the temporal relation of optimism to depression occurrence is not clear (i.e., higher optimism levels may precede or follow lower depressive symptoms), we lagged our analyses by 2 years to minimize the possibility of reverse causation. Because some items used to evaluate the constructs of optimism and depression may overlap, in sensitivity analyses, we excluded the CESD-10 item ‘*I felt hopeful about the future*’ and the GDS-15 item “*Do you feel that your situation is hopeless?*”; the presence of severe depressive symptoms was still defined using the same validated cutoffs, but without considering the aforementioned item in the scoring. Finally, to consider LOT-R’s potential bi-dimensionality [32], we investigated the three positively and three negatively worded items separately in relation to depression using one variable for each dimension.

All p-values were two-sided and considered statistically significant if p < 0.05. We used SAS software, version 9.4 (SAS Institute, Cary, North Carolina, United States) for all statistical analyses.

## Results

Participants were aged 57–85 (*mean* = 69.9, *SD* = 6.8), rather optimistic (*LOT-R: mean* = *19.9, SD* = *3.8*) and showed very low levels of depressive symptoms (*CESD-10: mean* = *4.0, SD* = *2.5*) at baseline. Optimism correlated with younger age (*Pearson´s r* = − *0.11*), lower levels of depressive symptoms (*r* = − *0.39*) and better physical functioning (*r* = *0.14*) at baseline. Compared to least optimistic women (bottom quartile), women who were more optimistic (top quartile) were more likely to not have been born in the northeast, to have received a higher education and a higher subjective societal position. Their husbands’ educational status was higher, and they were more likely to have had a father with a professional or managerial occupation. Women with higher optimism had also more social-emotional support, were more likely to be socially integrated and reported healthier behaviors. Retirement was more prevalent among less optimistic participants, as were negative health characteristics, including bodily pain and physical functioning (Table 1).

**Table 1:** Characteristics of Study Participants (*N* = 33,483) in the Nurses´ Health Study across optimism quartiles at Study Baseline in 2004

	Optimism quartiles			
	Q_1_ _(least optimistic)_ (*N* = 8,383)	Q_2_ (*N* = 7,941)	Q_3_ (*N* = 9,736)	Q_4_ _(most optimistic)_ (*N* = 7,423)
Optimism score	14.5 (2.3)	19.1 (0.8)	22.0 (0.8)	24 (0)
Range	*0–17*	*18–20*	*21–23*	*24–24*
CESD-10 depression score	5.3 (2.3)	4.3 (2.4)	3.7 (2.3)	2.6 (2.2)
*Demographic variables*				
Age^*^	71.1 (6.9)	70.3 (6.8)	69.2 (6.7)	68.9 (6.7)
Race^* a^				
Non-Hispanic white, %	93.6	94.4	94.8	94.8
Black, %	0.7	0.5	0.8	0.9
Others ^b^, %	5.7	5.1	4.4	4.3
Region of birth^* [a]^				
West, %	7.4	8.8	9.3	10.1
Midwest, %	23.9	24.7	25.6	25.5
Northeast, %	63.6	60.8	59.7	57.7
South, %	5.1	5.7	5.4	6.7
*Socio-economic variables*				
Highest education ^a^				
Registered nurse degree, %	74.6	69.2	65.3	62.9
Bachelor degree, %	18.1	20.9	22.3	23.3
Advanced degree, %	7.3	9.9	12.4	13.8
Subjective societal position ^c^				
High, %	9.6	12.7	16.6	23.2
Medium–high, %	51.1	57.4	59.4	56.9
Medium–low or low, %	39.3	29.9	24.0	20.0
Work status				
Retired, %	43.8	40.9	40.0	39.5
Marital status				
Married, %	70.1	72.2	72.1	72.2
Widowed, %	23.0	21.1	21.6	21.3
Other ^d^, %	6.9	6.7	6.3	6.5
Living arrangement				
With spouse, %	71.0	73.2	73.2	73.0
Alone, %	23.2	21.7	22.2	22.2
Other ^e^, %	5.8	5.2	4.7	4.8
Husband´s highest education ^a^				
≤ High school graduate, %	48.6	43.5	41.8	41.3
College graduate, %	28.9	30.7	30.1	30.8
Graduate school, %	22.5	25.8	28.1	27.9
Father´s occupation ^a^				
Professional/Managerial, %	23.5	25.6	27.6	29.2
Clerical/sales/service, %	38.5	39.1	38.8	38.2
Craftsmen/laborer/farmer, %	28.5	26.1	24.7	24.3
Other ^f^, %	9.5	9.2	8.9	8.3
Social-emotional support				
Communicate with confidant at least once per day, %	30.9	33.0	36.2	39.4
Weekly, %	43.9	44.9	44.0	43.2
Monthly, %	10.1	10.6	9.5	8.3
Several times per year, %	7.8	7.2	6.6	6.1
No confidant, %	7.3	4.3	3.7	3.0
Social network index				
Highly socially isolated, %	12.0	10.0	8.5	8.2
Moderately isolated, %	26.5	22.4	21.3	20.9
Moderately integrated, %	36.0	37.8	38.3	37.6
Highly socially integrated, %	25.5	29.8	31.9	33.3
Care for grandchildren ^c^				
No, %	66.7	67.4	68.1	69.5
Some, %	28.9	28.7	28.6	26.8
High, %	4.4	3.9	3.3	3.7
Care for disabled/ill person ^c^				
No, %	81.3	81.5	81.6	82.2
Some, %	13.6	13.5	13.4	12.9
High, %	5.1	5.0	5.0	4.9
*Lifestyle variables*				
Body mass index (BMI)	26.1 (5.1)	26.1 (5.0)	25.9 (4.9)	26.0 (4.8)
Normal weight (BMI < 25), %	46.4	47.5	49.9	48.3
Healthy physical activity ^g^, %	32.9	36.5	39.8	41.2
Non-smoker, %	93.2	94.2	94.4	95.0
Healthy alcohol consumption ^h,i^ %	19.7	21.9	23.8	23.2
Healthy diet ^i,j^, %	33.3	37.9	42.4	45.2
*Health depicting variables*				
Bodily pain ^c^				
None, %	16.6	17.8	19.9	25.3
Very mild/mild, %	62.0	63.8	62.6	60.7
Moderate, %	19.1	16.2	15.9	12.4
Severe/very severe, %	2.3	2.1	1.6	1.6
Problem falling asleep or maintaining sleep ^[c]^				
Most/all of the time, %	3.3	2.6	2.4	1.6
Good bit/some of the time, %	30.2	26.6	23.1	18.9
A little of the time, %	34.3	34.2	34.8	32.0
None of the time, %	32.2	36.6	39.7	47.5
Sleep duration ^k^				
< 7 h., %	26.6	23.1	20.4	20.1
7–8 h., %	67.3	70.6	73.3	72.9
> 8 h., %	6.1	6.3	6.3	7.0
Physical functioning score ^l^	74.1 (23.8)	76.6 (22.5)	78.4 (21.8)	80.5 (21.6)
Comorbidity burden ^m^, %	9.7	8.8	7.3	7.1
Minor tranquilizer use ^n^, %	3.8	3.1	2.6	2.4

Values are means(SD) or medians (Q25, Q75) for continuous variables; percentages for categorical variables, and are standardized to the age distribution of the study population. ^*^ Value is not age adjusted

[a] assessed in 1992

[b] any other race, e.g. Asian, American Indian

[c] assessed in 2000

[d] any other status, e.g. never married, divorced

[e] any other living arrangement, e.g. nursing home, with other family

[f] any other occupation, e.g. always working from home

[g] ≥ 150 min per week of moderate to vigorous activity

[h] 1 drink/day on average

[i] assessed in 2006

[j] score of the Alternative Healthy Eating Index (AHEI) in the top 40% of the current cohort distribution

[k] assessed in 2002

[l] higher scores indicate better functioning (*Range*: 0–100)

[m] ≥ 2 major chronic diseases

[n] Valium, Xanax, Ativan or Librium

During 10-years follow-up, we documented 4,051 incident depression cases (overall incidence 14.0 cases per 1,000 person-years). In age-adjusted models, baseline optimism levels were substantially and inversely associated with risk of depression (Table 2; Q1 vs. Q4 optimism score: HR = 0.46, 95%CI = 0.42–0.51). While women with pre-existing clinical depression or severe depressive symptoms were excluded at baseline, additionally adjusting for baseline low-to-moderate depressive symptoms score attenuated the effect estimates, although they remained meaningful (Q1 vs. Q4: HR = 0.71, 95%CI = 0.64–0.78). In fully-adjusted models, socioeconomic and health depicting covariates did not appear to be important confounders in these associations (Q1 vs. Q4: HR = 0.73, 95%CI = 0.66–0.81). When considering optimism continuously, every 1-SD increase of optimism was associated with a 15% (95%CI = 12%-18%) lower risk of depression in the fully-adjusted model (Table 2).

**Table 2:** Association of dispositional optimism and incident depression risk ^a^ in the Nurses´ Health Study (*N* = 33,483), 2004–2014

		Optimism quartiles				Increase of one *standard deviation* ^b^
		Q_1_ _(least optimistic)_ (*N* = 8,383)	Q_2_ (*N* = 7,941)	Q_3_ (*N* = 9,736)	Q_4_ _(most optimistic)_ (*N* = 7,423)	
Cases/person-years		1480/68781	1000/68133	958/86002	613/66476	
Incident rate per 1000 person-years		21.5	14.7	11.1	9.2	
**Model 1:** Age-adjusted model	HR (95% CI)	1	0.70 (0.65–0.76)	0.56 (0.51–0.60)	0.46 (0.42–0.51)	0.74 (0.72–0.76)
**Model 2:** Model1 + baseline depressive symptoms	HR (95% CI)	1	0.80 (0.74–0.87)	0.71 (0.65–0.77)	0.71 (0.64–0.78)	0.84 (0.81–0.87)
**Model 3:** Model2 + demographic covariates	HR (95% CI)	1	0.81 (0.74–0.87)	0.71 (0.66–0.78)	0.72 (0–65-0.80)	0.84 (0.82–0.87)
**Model 4:** Model2 + health depicting covariates	HR (95% CI)	1	0.82 (0.75–0.89)	0.73 (0.67–0.79)	0.73 (0.66–0.81)	0.85 (0.82–0.88)
**Model 5:** Fully-adjusted	HR (95% CI)	1	0.81 (0.75–0.88)	0.73 (0.67–0.79)	0.73 (0.66–0.81)	0.85 (0.82–0.88)

CI = confidence interval; HR = hazard ratio

[a] self-reported clinician diagnosis or a new regular use of antidepressants on biennial questionnaires or reporting clinical depressive symptoms according to the fifteen-item Geriatric Depression Scale (score ≥ 6)

[b] of the standard (z-) score distribution of the optimism scale

***Model 1*:** Age-adjusted [< 65; 65–70; 71–75; 76–80, > 80 years]

***Model 2*:** Model1 + adjusted for baseline depressive symptoms [continuous]

***Model 3*:** *Model 2* + adjusted for educational status [Registered nurse; Bachelor´s degree; Advanced degree], region of birth [West; Midwest; Northeast; South], race [Non-Hispanic white; black; other], subjective societal status [High; Medium–High; Medium–low or low], work status [Retired; Homemaker; Full/part time non nursing; Full/part time nursing], living arrangement [With spouse; Alone; Other], marital status [Married; Widowed; Other], husband´s educational status [High school graduate or less; College graduate; Graduate school] and father´s occupation [Professional or managerial; Clerical, sales or service; Other]

***Model 4*:** *Model 2* + adjusted for bodily pain [None; Very mild/mild; Moderate; Severe/very severe], physical functioning [continuous], sleep duration [< 7; 7–8; > 8 h.], problem falling asleep or maintaining sleep [None of the time; A little of the time; Some/good bit of the time; Most/All of the time], providing care for grandchildren [None; Some; High] or an ill/disabled person [None; Some; High], multiple comorbidity [< 2; ≥ 2 chronic diseases] and minor tranquilizers use [binary]

***Model 5*:** Includes all the covariates above

Defining depression as either clinician-diagnosed depression OR antidepressant use (cases: *N* = 2,739, 9.4 cases per 1000 person-years) largely attenuated the association in fully-adjusted models when using categorical optimism levels (Table 3; Q1 vs. Q4: HR = 0.93, 95%CI = 0.82–1.05), although the association remained evident when using a continuous exposure (fully-adjusted model, per 1-*SD* increase: HR = 0.94, 95%CI = 0.90–0.98). When defining depression as clinician-diagnosed depression AND antidepressants use (cases: *N* = 1,055, 2.9 cases per 1000 person-years), the association was no longer apparent or effect estimates did not reach significance in fully-adjusted models (Table 4; per 1-SD increase: HR = 0.97, 95%CI = 0.90–1.04).

**Table 3:** Association of dispositional optimism and incident depression risk in the Nurses´ Health Study (*N* = 33,483) in which depression was defined as either a self-reported diagnosis of depression OR self-reported antidepressant use, 2004–2014

		Optimism quartiles				Increase of one standard deviation ^a^
		Q_1_ _(least optimistic)_ (*N* = 8,383)	Q_2_ (*N* = 7,941)	Q_3_ (*N* = 9,736)	Q_4_ _(most optimistic)_ (*N* = 7,423)	
Cases/person-years		845/70788	702/68880	704/86646	488/66774	
Incident rate per 1000 person-years		11.9	10.2	8.1	7.3	
**Model 1:** Age-adjusted model	HR (95% CI)	1	0.86 (0.78–0.96)	0.71 (0.64–0.78)	0.64 (0.57–0.71)	0.83 (0.80–0.86)
**Model 2:** Model1 + baseline depressive symptoms	HR (95% CI)	1	0.98 (0.89–1.09)	0.87 (0.79–0.97)	0.93 (0.82–1.04)	0.94 (0.90–0.98)
**Model 3:** Model2 + demographic covariates	HR (95% CI)	1	0.97 (0.87–1.07)	0.86 (0.77–0.95)	0.91 (0.80–1.03)	0.93 (0.90–0.97)
**Model 4:** Model2 + health depicting covariates	HR (95% CI)	1	1.00 (0.90–1.10)	0.89 (0.80–0.99)	0.95 (0.84–1.07)	0.95 (0.91–0.99)
**Model 5:** Fully-adjusted	HR (95% CI)	1	0.98 (0.89–1.09)	0.87 (0.79–0.97)	0.93 (0.82–1.05)	0.94 (0.90–0.98)

Risk estimates are Hazard ratios (HR) with 95% Confidence intervals (CI)

[a] of the standard (z-) score distribution of the optimism scale

***Model.1:*** Age-adjusted [< 65; 65–70; 71–75; 76–80, > 80 years]

***Model.2:*** Additionally adjusted for baseline depressive symptoms [continuous]

***Model 3:**** Model 2* + adjusted for educational status [Registered nurse; Bachelor´s degree; Advanced degree], region of birth [West; Midwest; Northeast; South], race [Non-Hispanic white; black; other], subjective societal status [High; Medium–High; Medium–low or low], work status [Retired; Homemaker; Full/part time non nursing; Full/part time nursing], living arrangement [With spouse; Alone; Other], marital status [Married; Widowed; Other], husband´s educational status [High school graduate or less; College graduate; Graduate school] and father´s occupation [Professional or managerial; Clerical, sales or service; Other]

***Model 4:**** Model 2* + adjusted for bodily pain [None; Very mild/mild; Moderate; Severe/very severe], physical functioning [continuous], sleep duration [< 7; 7–8; > 8 h.], problem falling asleep or maintaining sleep [None of the time; A little of the time; Some/good bit of the time; Most/All of the time], providing care for grandchildren [None; Some; High] or an ill/disabled person [None; Some; High], multiple comorbidity [< 2; ≥ 2 chronic diseases] and minor tranquilizers use [binary]

***Model 5*:** Includes all the covariates above

**Table 4:** Association of dispositional optimism and incident depression risk in the Nurses´ Health Study (*N* = 33,483) in which depression was defined as a self-reported diagnosis of depression AND self-reported antidepressants use, 2004–2014

		Optimism quartiles				Increase of one standard deviation ^a^
		Q_1_ _(least optimistic)_ (*N* = 8,383)	Q _2_ (*N* = 7,941)	Q_3_ (*N* = 9,736)	Q_4_ _(most optimistic)_ (*N* = 7,423)	
Cases/person-years		266/71377	221/69353	211/87139	158/67102	
Incident rate per 1000 person-years		3.7	3.2	2.4	2.3	
**Model 1:** Age-adjusted model	HR (95% CI)	1	0.86 (0.72–1.03)	0.66 (0.55–0.79)	0.62 (0.51–0.76)	0.83 (0.78–0.88)
**Model 2:** Model1 + baseline depressive symptoms	HR (95% CI)	1	1.01 (0.84–1.20)	0.86 (0.72–1.04)	0.99 (0.80–1.23)	0.96 (0.90–1.03)
**Model 3:** Model2 + demographic covariates	HR (95% CI)	1	1.00 (0.83–1.20)	0.86 (0.71–1.04)	0.99 (0.80–1.23)	0.96 (0.90–1.03)
**Model 4:** Model2 + health depicting covariates	HR (95% CI)	1	1.02 (0.85–1.22)	0.88 (0.73–1.06)	1.01 (0.82–1.26)	0.97 (0.91–1.04)
**Model 5:** Fully-adjusted	HR (95% CI)	1	1.01 (0.84–1.21)	0.87 (0.72–1.05)	1.01 (0.81–1.26)	0.97 (0.90–1.04)

Risk estimates are Hazard ratios (HR) with 95% Confidence intervals (CI)

[a] of the standard (z-) score distribution of the optimism scale

***Model.1:*** Age-adjusted [< 65; 65–70; 71–75; 76–80, > 80 years]

***Model.2:*** Additionally adjusted for baseline depressive symptoms [continuous]

***Model 3:**** Model 2* + adjusted for educational status [Registered nurse; Bachelor´s degree; Advanced degree], region of birth [West; Midwest; Northeast; South], race [Non-Hispanic white; black; other], subjective societal status [High; Medium–High; Medium–low or low], work status [Retired; Homemaker; Full/part time non nursing; Full/part time nursing], living arrangement [With spouse; Alone; Other], marital status [Married; Widowed; Other], husband´s educational status [High school graduate or less; College graduate; Graduate school] and father´s occupation [Professional or managerial; Clerical, sales or service; Other]

***Model 4:**** Model 2* + adjusted for bodily pain [None; Very mild/mild; Moderate; Severe/very severe], physical functioning [continuous], sleep duration [< 7; 7–8; > 8 h.], problem falling asleep or maintaining sleep [None of the time; A little of the time; Some/good bit of the time; Most/All of the time], providing care for grandchildren [None; Some; High] or an ill/disabled person [None; Some; High], multiple comorbidity [< 2; ≥ 2 chronic diseases] and minor tranquilizers use [binary]

***Model 5*:** Includes all the covariates above

Being socially integrated, receiving social-emotional support and adopting a healthy lifestyle mediated the association of optimism with future depression risk (sTable 2). Jointly they accounted for about 10.2% (95%CI = 7.3%-14.3%) of the lower depression risk found among the women in the top versus bottom quartile of optimism.

Across groups with different baseline depressive symptom levels (sTable 3), optimism was similarly associated with a lower depression risk. When applying the more restrictive depression definitions the association appeared to be slightly stronger among participants with higher baseline depressive symptoms although some strata had a small number of cases (e.g., n = 30) (sTables 4–5). However, the relationship was similar across all age groups (sTable 6) and birth regions (sTable 7). Although the reduced risk was slightly smaller among Non-Hispanic Whites than among other racial groups combined, the likelihood ratio test was not statistically significant **(**e.g., Model 1, *p* = 0.391), suggesting that the optimism-depression relationship was comparable across race (sTable 8).

When implementing a 2-year lag time between optimism and depression incidence, effect estimates were slightly stronger (e.g., in fully-adjusted model: Q1 vs. Q4: HR = 0.70, 95%CI = 0.62–0.78; sTable 9). When trying to disentangle the conceptual overlap between the exposure and the outcome, not including the item *“I felt hopeful about the future”* of the CESD-10 when adjusting for baseline depressive symptoms lead to slightly stronger effect estimates (sTable 10). Not considering the item “*Do you feel that your situation is hopeless*” for scoring in the GDS, finally, did not affect estimates (sTable 11). The separate analysis of the three positively and three negatively worded items of the LOT-R indicated that both being more optimistic and being less pessimistic were associated with a lower depression risk (sTable 12).

## Discussion

In the present study, older women who reported higher versus lower optimism at baseline had a reduced risk of incident depression throughout 10 years of follow-up, after adjustment for a wide array of potentially relevant covariates including baseline mild depressive symptoms. This association was evident irrespective of age, race and region of residence, and after lagging analyses by 2 years to reduce concerns about reverse causation. Mediation models suggested that lifestyle and social factors explained partly but not fully the association of optimism with depression risk. Applying more restrictive depression definitions that excluded self-reported severe depressive symptoms from the outcome measure, however, revealed attenuated or null estimates.

Results from primary models are in line with our hypotheses, based on findings from other prospective studies though comparability is somewhat limited due to differences in exposure and outcome assessment [7–10]. For instance, in the Zutphen Elderly Study, high vs. low optimism, assessed with a 4-item validated scale, predicted a lower cumulative incidence of depressive symptoms, as defined by a validated self-reported measure [10]. In one of the Finish Public Sector studies, higher versus lower optimism was associated with a reduced likelihood of starting antidepressant medication (HR = 0.67, 95%CI = 0.62–0.73) and a greater likelihood of stopping antidepressant use (HR = 1.18, 95%CI = 1.08–1.30) [7]. In the same cohort, higher optimism was associated with lower likelihood of initiating psychotherapy as a treatment for depression (HR = 0.57, 95%CI = 0.40–0.81); in 38,717 participants of the Finish Public Sector study the authors also found a lower likelihood of depressive disorder (HR = 0.68, 95%CI = 0.62–0.73) based on purchase of antidepressants, long-term work disability or hospitalization due to depression and after accounting for sex, age, marital status, socioeconomic position, alcohol consumption, anxiolytics and hypnotics purchase, and chronic medical conditions [8]. In other studies, optimism predicted long-term work disability with a diagnosis of depression, and the likelihood of returning to work [9]. The outcomes defined within the Finish Public Sector Study were similar to our more restrictive depression definitions. However, that they did not control for baseline depression may have rendered their results biased given that in our study, associations were attenuated, although still meaningful, after adjustment for baseline depressive symptoms.

That effect estimates were attenuated (more restrictive definition) or disappeared (most restrictive definition) in our study when using more restrictive definitions of depression (i.e., higher specificity for identifying depression) may suggest that optimism might serve as a protective factor for mild or moderate depression, but not for higher severity clinical depression. Alternatively, such attenuation in estimates may also be explained by measurement issues: associations could be stronger for self-reported depressive symptoms score because of greater variability compared to binary diagnosis/medication and allow the identification of additional depression cases (e.g. depressive participants who would not seek clinician’s help or be prescribed medication). Lastly, some optimism and depression attributes overlap conceptually; yet, estimates were stable when removing items that could characterize both optimism and depression.

We found no evidence that the association would differ importantly across baseline depressive symptoms, age, race, or region of birth. The majority of existing studies did not consider stratified analyses, except by age, with two previous studies reporting that the association of optimism and depression risk would wane with increasing age when examined over a broader age range than our study [33, 34].

Several explanations exist as to why optimists might be less prone to develop depression. First, optimism and depression might share a common genetic disposition, potentially explaining a third of the phenotypic association between them [35]. Second, childhood adversity [36] could account for the association, whereby younger individuals exposed to major stressors early in life would be more likely to develop depression and less likely to maintain an positive outlook on life. To our knowledge, no previous study has examined this hypothesis. Third, optimists were shown to maintain a healthier lifestyle [15], receive more social support [13, 14] and apply more effective coping strategies [12]. For instance, optimists are more likely to recognize and disengage from unsolvable problems and are therefore able to focus their energy on situations that are solvable, making them potentially less likely to experience avoidable disappointments [11]. In our study, the association between optimism and depression incidence was modestly mediated by social network, social-emotional support and a healthy lifestyle but these three factors individually or jointly explained only a small portion of the reported effect. Other factors, including alternative coping strategies (e.g., planning, problem-solving), might be of greater importance and should be considered in future research.

The principal strength of our study is the control of most known determinants of depression and optimism, including baseline depressive symptoms. Additional strengths are the prospective study design with 10-year follow-up, validated optimism assessment, having 3 indicators of depression, and a large number of participants, affording sufficient statistical power even for sensitivity analyses that verified the robustness of the optimism-depression association. However, because the NHS represents a highly homogenous sample of mostly white women who were all nurses, results might not be generalizable to other populations, including women in non-medical occupations and men, although incident depression rates in the Nurses’ cohort have been found to be highly consistent with expected age- and sex/gender-specific rates. Though nurses, especially experienced ones, might be considered more resilient compared to women from the general population, their major depression prevalence is roughly comparable (8.7%) [37, 38] The generalizability of our results might be reduced by high attrition rates in the study; women with missing information on optimism or depressive symptoms differed importantly from the analytic sample. Further, we had no information on other protective factors that might characterize optimistic individuals (e.g., coping strategies like planning and problem-solving) and in turn, influence depression risk, to examine their potential mediating role. Finally, although we excluded women with any depressive episode prior to or at baseline, it is likely that we did not capture all participants with a prior depressive episode since an important proportion of mood disorders have already developed by mid-adolescence [39]. Depressive symptoms might cause persistent scars in personality traits [40], such as optimism, and therefore part of our results could be explained by reverse causality.

Optimism as a potential modifiable determinant of depression risk in late life may be promising based on prior intervention research. Currently, the Best Possible Self exercise is considered the most effective strategy to increase dispositional optimism. Briefly, one imagines a future possible self, pictures this possible self and positive future situations in detail, and then creates a mental plan how to achieve this imagined self [17]. A recent meta-analysis [41] indicates that Best Possible Self interventions are effective in increasing optimism outcomes although evidence on long-term effects is currently still insufficient; and only a small non-significant effect on depressive symptoms was reported based on three interventions in young participants with one to three months of follow-up [41–44]. Long term effects on depressive symptoms, particularly in midlife and older adults, remain unknown.

Several aspects of the association between dispositional optimism and depression are still to be resolved. Future prospective studies are needed to evaluate the association between optimism and depression risk in various populations while considering other protective factors of resilience and controlling for shared genetic dispositions and childhood environment factors. Further, better understanding how and when psychological protective characteristics like optimism develop and consolidate over the life course is warranted, to eventually guide intervention research toward specific time windows that appear more potent to prevent depression risk in later life. Evidence suggests that optimism levels would be relatively similar across age groups [33] although they may start to decline around late midlife [45, 46]. Therefore, if optimism-enhancing interventions truly protect against subsequent depression risk, future clinical trials aiming to reduce the depression burden among the elderly might be ideally implemented before optimism levels start declining.

## Supplementary Information

Below is the link to the electronic supplementary material.

Supplementary file1 (DOCX 72 kb)

## Data Availability

"Further information including the procedures to obtain and access data from the Nurses’ Health Studies and Health Professionals Follow-up Study is described at https://www.nurseshealthstudy.org/researchers (contact email: nhsaccess@channing.harvard.edu) and https://sites.sph.harvard.edu/hpfs/for-collaborators/."
