# Supplementary material for: Dispositional optimism and depression risk in older women in the Nurses´ Health Study: a prospective cohort study
Source: Eur J Epidemiol. 2022 Jan 15;37(3):283–94. doi: 10.1007/s10654-021-00837-2 (PMC9110484; doi:10.1007/s10654-021-00837-2)
Supplement: Supplementary file 1 — Supplementary file1 (DOCX 72 kb) [file 10654_2021_837_MOESM1_ESM.docx]

| **Supplementary table 1.** Comparison of characteristics of the analytic sample and participants with missing information on dispositional optimism who were excluded at baseline ^a^ | | | |
| --- | --- | --- | --- |
|  | Analytic sample  (*N*=33,483) | Participants with missing information on dispositional optimism ^a^  (*N*=1,221) | Participants with any missing information on depressive symptoms before or at study baseline (*N=37,002*) |
| Age | 68.9 (6.8) | 71.6 (6.9) | 69.5 (7.1) |
| Race ^b^ |  |  |  |
| Non-Hispanic white, % | 94.4 | 92.3 | 91.2 |
| Black, % | 0.7 | 1.2 | 2.7 |
| Others ^c^, % | 4.9 | 6.5 | 6.1 |
| Region of birth ^b^ |  |  |  |
| West, % | 8.9 | 9.5 | 9.7 |
| Midwest, % | 24.9 | 23.1 | 21.8 |
| Northeast, % | 60.5 | 60.1 | 61.4 |
| South, % | 5.7 | 7.3 | 7.1 |
| Highest education ^b^ |  |  |  |
| Registered nurse degree, % | 68.0 | 74.2 | 80.6 |
| Bachelor degree, % | 21.1 | 17.4 | 13.3 |
| Advanced degree, % | 10.9 | 8.4 | 6.1 |
| Work status |  |  |  |
| Retired, % | 41.1 | 45.7 | 44.3 |
| Marital status |  |  |  |
| Married, % | 71.6 | 69.0 | 66.7 |
| Widowed, % | 21.8 | 25.4 | 23.5 |
| Other ^d^, % | 6.6 | 5.6 | 9.8 |
| Physical functioning score ^e^ | 77.4 (22.6) | 73.2 (25.5) | 70.8 (26.8) |
| Comorbidity burden ^f^, % | 8.1 | 11.2 | 13.1 |
| Values are means(SD) or medians (Q25, Q75) for continuous variables; percentages for categorical variables.  [a] after the exclusion of individuals who fell into one of the first three exclusion criteria (See.Figure.1)  [b] assessed in 1992  [c] any other race, e.g. Asian, American Indian  [d] any other status, e.g. never married, divorced  [e] higher scores indicate better functioning (*Range*: 0-100)  [f] ≥2 major chronic diseases | | |  |

| **Supplementary table 2**. Estimated proportion of association between dispositional optimism and incident depression ^a^ risk in the Nurses’ Health Study (*N*= 33,483) explained by social network size, social emotional support and lifestyle ^b^ | | | | |  |
| --- | --- | --- | --- | --- | --- |
|  | Optimism quartiles | | | | |
|  | Q_1_  _(least optimistic)_  (*N* = 8,383) | Q_2_  (*N*= 7,941) | Q_3_  (*N* = 9,736) | Q_4_  _(most optimistic)_  (*N*= 7,423) | |
| **Mediator adjusted model** | 1 | 0.82 (0.76-0.89) | 0.74 (0.69-0.81) | 0.76 (0.70-0.82) | |
| **Mediator 1: Social network size** | |  |  |  | |
| HR (95% CI) ^c^ | 1 | 0.83 (0.76-0.90) | 0.74 (0.68-0.80) | 0.75 (0.69-0.81) | |
| Proportion mediated |  | **3.6% (1.8-7.2)** | **3.9% (2.5-5.9)** | **4.2% (2.7-6.4)** | |
| **Mediator 2: Social-emotional support** | |  |  |  | |
| HR (95% CI) ^d^ | 1 | 0.83 (0.76-0.89) | 0.74 (0.68-0.81) | 0.75 (0.69-0.82) | |
| Proportion mediated |  | **<1%** | **<1%** | **1.2% (0.5-3.2)** | |
| **Mediator 3: Healthy lifestyle** | |  |  |  | |
| HR (95% CI) ^e^ | 1 | 0.82 (0.76-0.89) | 0.74 (0.68-0.80) | 0.75 (0.69-0.81) | |
| Proportion mediated |  | **1.8% (0.5-6.3)** | **2.3% (1.2-4.4)** | **4.2% (2.7-6.4)** | |
| **Mediators 1, 2 and 3 combined** |  |  |  |  | |
| HR (95% CI) ^f^ | 1 | 0.81 (0.75-0.88) | 0.73 (0.67-0.79) | 0.73 (0.66-0.81) | |
| Proportion mediated |  | **7.2% (3.9-13.1)** | **7.9% (5.4-11.4)** | **10.2% (7.3-14.3)** | |
| CI=confidence interval; HR=hazard ratio.  [a] self-reported physician/clinician diagnosis or a new regular use of antidepressants on biennial questionnaires or reporting clinical depressive symptoms according to the fifteen-item Geriatric Depression Scale (score≥6)  ***Mediator adjusted model:*** *Model 5 (see table 2)* + adjusted for all following potential mediators: social network size [Socially isolated; Moderately isolated; Moderately integrated; Socially integrated], social emotional support [Communicate with confidant at least once per day; Weekly; Monthly; Several times per year; No confidant] and healthy lifestyle variables (BMI [<25 kg/m2; ≥25 kg/m2]; Not currently smoking [Binary]; Healthy physical activity [≥150 minutes per week of moderate-to vigorous activity]; Healthy diet [Total score of the Alternative Healthy Eating Index (AHEI) in the top 40% of the current cohort distribution] and Healthy alcohol consumption [Drinking 1 drink/day on average]).  [b] Mediators were assessed at baseline and updated each 2-4 years in the models. The mediator adjusted model was compared to the same model without adjustment for the mediator to estimate the mediated proportion [c] not adjusted for social network size [d] not adjusted for social-emotional support [e] not adjusted for lifestyle variables [f] not adjusted for any of the three mediators | | | | | |

| **Supplementary table 3**. Association of dispositional optimism and risk of incident depression ^a^ in the Nurses´ Health Study (*N*= 33,483) across depressive symptoms levels at baseline (2004), 2004-2014. | | | | |
| --- | --- | --- | --- | --- |
|  |  | Baseline depressive symptoms | | |
|  |  | Very low ^b^  (*N*= 10,487) | Low ^c^  (*N*= 13,373) | Moderate ^d^  (*N*= 9,623) |
| Cases/person-years |  | 707/94,566 | 1,502/116,535 | 1,842/78,286 |
| Incident rate per 1000 person-years | | 7.5 | 12.9 | 23.5 |
| **Optimism quartiles** |  |  |  |  |
| First | **Model 1:** age-adjusted | 1 | 1 | 1 |
| Second |  | 0.80 (0.62-1.04) | 0.76 (0.67-0.87) | 0.80 (0.72-0.89) |
| Third |  | 0.71 (0.56-0.90) | 0.67 (0.58-0.76) | 0.69 (0.61-0.78) |
| Fourth |  | 0.70 (0.55-0.88) | 0.70 (0.60-0.81) | 0.58 (0.48-0.71) |
| HR (95% CI) for an increase of one *SD* ^e^ | | 0.86 (0.79-0.94) | 0.83 (0.79-0.88) | 0.81 (0.77-0.84) |
| First | **Model 2:** Model1 + baseline depressive symptoms | 1 | 1 | 1 |
| Second |  | 0.82 (0.63-1.06) | 0.78 (0.68-0.89) | 0.82 (0.73-0.92) |
| Third |  | 0.73 (0.57-0.93) | 0.70 (0.61-0.80) | 0.72 (0.63-0.81) |
| Fourth |  | 0.75 (0.59-0.95) | 0.75 (0.64-0.88) | 0.62 (0.51-0.75) |
| HR (95% CI) for an increase of one *SD* ^e^ | | 0.88 (0.81-0.96) | 0.85 (0.81-0.90) | 0.82 (0.78-0.86) |
| First | **Model 3:** fully-adjusted | 1 | 1 | 1 |
| Second |  | 0.82 (0.63-1.06) | 0.80 (0.70-0.92) | 0.83 (0.74-0.93) |
| Third |  | 0.74 (0.58-0.95) | 0.72 (0.63-0.83) | 0.73 (0.65-0.83) |
| Fourth |  | 0.75 (0.59-0.95) | 0.78 (0.67-0.92) | 0.64 (0.53-0.78) |
| HR (95% CI) for an increase of one *SD* ^e^ | | 0.89 (0.81-0.97) | 0.87 (0.82-0.91) | 0.84 (0.80-0.87) |

Risk estimates are Hazard ratios (HR) with 95% Confidence intervals (CI)

[a] self-reported physician/clinician diagnosis or a new regular use of antidepressants on biennial questionnaires or reporting clinical depressive symptoms according to the fifteen-item Geriatric Depression Scale (score≥6)

[b] 10-item Center for Epidemiological Studies Depression Scale (CESD-10) score <3

[c] 2< CESD-10 score <6

[d] 5< CESD-10 score <10

[e] of the standard (z-) score distribution of the optimism scale

***Model.1:*** Age-adjusted [<65; 65-70; 71-75; 76-80, >80 years]

***Model.2:*** Additionally adjusted for baseline depressive symptoms [continuous]

***Model 3:*** *Model 2* + adjusted for educational status [Registered nurse; Bachelor´s degree; Advanced degree], region of birth [West; Midwest; Northeast; South], race [Non-Hispanic white; black; other], subjective societal status [High; Medium-High; Medium-low or low], work status [Retired; Homemaker; Full/part time non nursing; Full/part time nursing], living arrangement [With spouse; Alone; Other], marital status [Married; Widowed; Other], husband´s educational status [High school graduate or less; College graduate; Graduate school] and father´s occupation [Professional or managerial; Clerical, sales or service; Other], bodily pain [None; Very mild/mild; Moderate; Severe/very severe], physical functioning [continuous], sleep duration [<7; 7-8; >8 hrs.], problem falling asleep or maintaining sleep [None of the time; A little of the time; Some/good bit of the time; Most/All of the time], providing care for grandchildren [None; Some; High] or an ill/disabled person [None; Some; High], multiple comorbidity [<2; ≥2 chronic diseases] and minor tranquilizers use [binary]

| **Supplementary table 4**. Association of dispositional optimism and risk of incident depression in the Nurses´ Health Study (*N*= 33,483) in which depression was defined as either a self-reported diagnosis of depression OR self-reported antidepressants use, across depressive symptoms levels at baseline (2004), 2004-2014. | | | | |
| --- | --- | --- | --- | --- |
|  |  | Baseline depressive symptoms | | |
|  |  | Very low ^a^  (*N*= 10,487) | Low ^b^  (*N*= 13,373) | Moderate ^c^  (*N*= 9,623) |
| Cases/person-years |  | 546/94,941 | 1058/117,686 | 1135/80,457 |
| Incident rate per 1000 person-years | | 5.8 | 9.0 | 14.1 |
| **Optimism quartiles** |  |  |  |  |
| First | **Model 1:** age-adjusted | 1 | 1 | 1 |
| Second |  | 1.04 (0.76-1.43) | 0.87 (0.74-1.02) | 1.00 (0.87-1.15) |
| Third |  | 0.91 (0.67-1.22) | 0.78 (0.66-0.91) | 0.88 (0.76-1.03) |
| Fourth |  | 0.92 (0.69-1.23) | 0.88 (0.73-1.05) | 0.81 (0.64-1.02) |
| HR (95% CI) for an increase of one *SD* ^d^ | | 0.94 (0.84-1.04) | 0.92 (0.86-0.98) | 0.92 (0.87-0.97) |
| First | **Model 2:** Model1 + baseline depressive symptoms | 1 | 1 | 1 |
| Second |  | 1.07 (0.78-1.47) | 0.89 (0.76-1.05) | 1.03 (0.89-1.18) |
| Third |  | 0.93 (0.70-1.25) | 0.81 (0.69-0.95) | 0.92 (0.78-1.07) |
| Fourth |  | 1.01 (0.75-1.34) | 0.94 (0.78-1.13) | 0.85 (0.67-1.07) |
| HR (95% CI) for an increase of one *SD* ^d^ | | 0.96 (0.87-1.07) | 0.94 (0.88-1.00) | 0.94 (0.89-0.99) |
| First | **Model 3:** fully-adjusted | 1 | 1 | 1 |
| Second |  | 1.06 (0.77-1.46) | 0.91 (0.77-1.07) | 1.02 (0.88-1.18) |
| Third |  | 0.93 (0.69-1.25) | 0.83 (0.70-0.98) | 0.90 (0.77-1.06) |
| Fourth |  | 0.99 (0.74-1.33) | 0.96 (0.79-1.15) | 0.85 (0.67-1.07) |
| HR (95% CI) for an increase of one *SD* ^d^ | | 0.96 (0.86-1.07) | 0.94 (0.88-1.01) | 0.94 (0.88-0.99) |

Risk estimates are Hazard ratios (HR) with 95% Confidence intervals (CI)

[a] 10-item Center for Epidemiological Studies Depression Scale (CESD-10) score <3

[b] 2< CESD-10 score <6

[c] 5< CESD-10 score <10

[d] of the standard (z-) score distribution of the optimism scale

***Model.1:*** Age-adjusted [<65; 65-70; 71-75; 76-80, >80 years]

***Model.2:*** Additionally adjusted for baseline depressive symptoms [continuous]

***Model 3:*** *Model 2* + adjusted for educational status [Registered nurse; Bachelor´s degree; Advanced degree], region of birth [West; Midwest; Northeast; South], race [Non-Hispanic white; black; other], subjective societal status [High; Medium-High; Medium-low or low], work status [Retired; Homemaker; Full/part time non nursing; Full/part time nursing], living arrangement [With spouse; Alone; Other], marital status [Married; Widowed; Other], husband´s educational status [High school graduate or less; College graduate; Graduate school] and father´s occupation [Professional or managerial; Clerical, sales or service; Other], bodily pain [None; Very mild/mild; Moderate; Severe/very severe], physical functioning [continuous], sleep duration [<7; 7-8; >8 hrs.], problem falling asleep or maintaining sleep [None of the time; A little of the time; Some/good bit of the time; Most/All of the time], providing care for grandchildren [None; Some; High] or an ill/disabled person [None; Some; High], multiple comorbidity [<2; ≥2 chronic diseases] and minor tranquilizers use [binary]

| **Supplementary table 5**. Association of dispositional optimism and risk of incident depression in the Nurses´ Health Study (*N*= 33,483) in which depression was defined as a self-reported diagnosis of depression AND self-reported antidepressants use across depressive symptoms levels at baseline (2004), 2004-2014. | | | |
| --- | --- | --- | --- |
|  |  | Baseline depressive symptoms | |
|  |  | Very low ^a^ or low ^b^  (*N*= 23,860) | Moderate ^c^  (*N*= 9,623) |
| Cases/person-years |  | 480/ 213,733 | 371/81,235 |
| Incident rate per 1000 person-years | | 2.2 | 4.6 |
| **Optimism quartiles** |  |  |  |
| First | **Model 1:** age-adjusted | 1 | 1 |
| Second |  | 0.86 (0.66-1.12) | 1.03 (0.81-1.32) |
| Third |  | 0.77 (0.59-0.99) | 0.77 (0.58-1.02) |
| Fourth |  | 0.76 (0.59-0.99) | 0.85 (0.58-1.26) |
| HR (95% CI) for an increase of one *SD* ^d^ | | 0.90 (0.82-0.98) | 0.91 (0.83-1.00) |
| First | **Model 2:** Model1 + baseline depressive symptoms | 1 | 1 |
| Second |  | 0.93 (0.71-1.21) | 1.08 (0.84-1.37) |
| Third |  | 0.88 (0.68-1.14) | 0.82 (0.62-1.09) |
| Fourth |  | 1.01 (0.77-1.33) | 0.94 (0.63-1.39) |
| HR (95% CI) for an increase of one *SD* ^d^ | | 0.99 (0.89-1.09) | 0.94 (0.85-1.04) |
| First | **Model 3:** fully-adjusted | 1 | 1 |
| Second |  | 0.94 (0.72-1.23) | 1.10 (0.86-1.40) |
| Third |  | 0.88 (0.68-1.14) | 0.83 (0.63-1.11) |
| Fourth |  | 1.01 (0.76-1.33) | 1.01 (0.68-1.50) |
| HR (95% CI) for an increase of one *SD* ^d^ | | 0.99 (0.89-1.09) | 0.96 (0.87-1.06) |

Risk estimates are Hazard ratios (HR) with 95% Confidence intervals (CI)

[a] 10-item Center for Epidemiological Studies Depression Scale (CESD-10) score <3

[b] 2< CESD-10 score <6

[c] 5< CESD-10 score <10

[d] of the standard (z-) score distribution of the optimism scale

***Model.1:*** Age-adjusted [<65; 65-70; 71-75; 76-80, >80 years]

***Model.2:*** Additionally adjusted for baseline depressive symptoms [continuous]

***Model 3:*** *Model 2* + adjusted for educational status [Registered nurse; Bachelor´s degree; Advanced degree], region of birth [West; Midwest; Northeast; South], race [Non-Hispanic white; black; other], subjective societal status [High; Medium-High; Medium-low or low], work status [Retired; Homemaker; Full/part time non nursing; Full/part time nursing], living arrangement [With spouse; Alone; Other], marital status [Married; Widowed; Other], husband´s educational status [High school graduate or less; College graduate; Graduate school] and father´s occupation [Professional or managerial; Clerical, sales or service; Other], bodily pain [None; Very mild/mild; Moderate; Severe/very severe], physical functioning [continuous], sleep duration [<7; 7-8; >8 hrs.], problem falling asleep or maintaining sleep [None of the time; A little of the time; Some/good bit of the time; Most/All of the time], providing care for grandchildren [None; Some; High] or an ill/disabled person [None; Some; High], multiple comorbidity [<2; ≥2 chronic diseases] and minor tranquilizers use [binary]

| **Supplementary table 6**. Association of dispositional optimism and risk of incident depression ^a^ in the Nurses´ Health Study (*N*= 33,483,) across age categories at baseline (2004), 2004-2014. | | | | |
| --- | --- | --- | --- | --- |
|  |  | Age | | |
|  |  | <65 years  (*N*= 8,648) | 65-74 years  (*N*= 15,189) | >74 years  (*N*= 9,646) |
| Cases/person-years |  | 820/80114 | 1708/134700 | 1523/74577 |
| Incident rate per 1000 person-years | | 10.2 | 12.7 | 20.4 |
| **Optimism quartiles** |  |  |  |  |
| First | **Model 1:** age-adjusted | 1 | 1 | 1 |
| Second |  | 0.72 (0.60-0.87) | 0.72 (0.58-0.75) | 0.72 (0.64-0.82) |
| Third |  | 0.52 (0.43-0.62) | 0.55 (0.49-0.62) | 0.59 (0.51-0.67) |
| Fourth |  | 0.48 (0.39-0.58) | 0.42 (0.47-0.49) | 0.50 (0.43-0.59) |
| HR (95% CI) for an increase of one *SD* ^b^ | | 0.74 (0.70-0.78) | 0.73 (0.70-0.76) | 0.75 (0.72-0.79) |
| First | **Model 2:** Model1 + baseline depressive symptoms | 1 | 1 | 1 |
| Second |  | 0.83 (0.69-1.00) | 0.77 (0.68-0.87) | 0.82 (0.72-0.93) |
| Third |  | 0.66 (0.55-0.80) | 0.71 (0.63-0.81) | 0.72 (0.63-0.83) |
| Fourth |  | 0.73 (0.59-0.90) | 0.67 (0.57-0.78) | 0.74 (0.63-0.88) |
| HR (95% CI) for an increase of one *SD* ^b^ | | 0.84 (0.79-0.90) | 0.84 (0.80-0.88) | 0.85 (0.80-0.89) |
| First | **Model 3**: fully-adjusted | 1 | 1 | 1 |
| Second |  | 0.87 (0.72-1.06) | 0.79 (0.69-0.89) | 0.82 (0.72-0.94) |
| Third |  | 0.70 (0.57-0.84) | 0.75 (0.66-0.85) | 0.74 (0.64-0.85) |
| Fourth |  | 0.77 (0.62-0.96) | 0.70 (0.60-0.82) | 0.76 (0.64-0.90) |
| HR (95% CI) for an increase of one *SD* ^b^ | | 0.85 (0.80-0.91) | 0.85 (0.81-0.89) | 0.86 (0.81-0.90) |

Risk estimates are Hazard ratios (HR) with 95% Confidence intervals (CI)

[a] self-reported physician/clinician diagnosis or a new regular use of antidepressants on biennial questionnaires or reporting clinical depressive symptoms according to the fifteen-item Geriatric Depression Scale (score≥6)

[b] of the standard (z-) score distribution of the optimism scale

***Model.1:*** Age-adjusted [<65; 65-70; 71-75; 76-80, >80 years]

***Model.2:*** Additionally adjusted for baseline depressive symptoms [continuous]

***Model 3:*** *Model 2* + adjusted for educational status [Registered nurse; Bachelor´s degree; Advanced degree], region of birth [West; Midwest; Northeast; South], race [Non-Hispanic white; black; other], subjective societal status [High; Medium-High; Medium-low or low], work status [Retired; Homemaker; Full/part time non nursing; Full/part time nursing], living arrangement [With spouse; Alone; Other], marital status [Married; Widowed; Other], husband´s educational status [High school graduate or less; College graduate; Graduate school] and father´s occupation [Professional or managerial; Clerical, sales or service; Other], bodily pain [None; Very mild/mild; Moderate; Severe/very severe], physical functioning [continuous], sleep duration [<7; 7-8; >8 hrs.], problem falling asleep or maintaining sleep [None of the time; A little of the time; Some/good bit of the time; Most/All of the time], providing care for grandchildren [None; Some; High] or an ill/disabled person [None; Some; High], multiple comorbidity [<2; ≥2 chronic diseases] and minor tranquilizers use [binary]

| **Supplementary table 7**. Association of dispositional optimism and risk of incident depression ^a^ in the Nurses´ Health Study (*N*= 32,204) ^b^ across birth region, follow-up 2004-2014. | | | | | | |
| --- | --- | --- | --- | --- | --- | --- |
|  | |  | Region of birth ^b^ | | |  |
|  | |  | West ^c^  (*N*= 2,859) | Midwest ^d^  (*N*= 8,032) | Northeast ^e^  (*N*= 19,489) | South ^f^  (*N*= 1,833) |
| Cases/person-years | |  | 330/24427 | 1000/69237 | 2317/169054 | 229/15678 |
| Depression incident rate per 1000 person-years | | | 13.5 | 14.4 | 13.7 | 14.6 |
| **Optimism quartiles** | |  |  |  |  |  |
| Q_1 (least optimistic)_ | | **Model 1** | 1 | 1 | 1 | 1 |
| Q_2_ | |  | 0.62 (0.46-0.82) | 0.71 (0.60-0.84) | 0.69 (0.62-0.76) | 0.74 (0.52-1.04) |
| Q_3_ | |  | 0.50 (0.38-0.66) | 0.52 (0.44-0.61) | 0.57 (0.51-0.63) | 0.50 (0.35-0.72) |
| Q_4 (most optimistic)_ | |  | 0.36 (0.26-0.50) | 0.52 (0.43-0.62) | 0.45 (0.40-0.51) | 0.44 (0.30-0.64) |
| HR (95% CI) for an increase of one *SD* ^g^ | | | 0.69 (0.62-0.76) | 0.75 (0.71-0.80) | 0.74 (0.71-0.76) | 0.73 (0.65-0.81) |
| Q_1 (least optimistic)_ | | **Model 2** | 1 | 1 | 1 | 1 |
| Q_2_ | |  | 0.71 (0.53-0.95) | 0.78 (0.66-0.92) | 0.81 (0.72-0.90) | 0.87 (0.61-1.23) |
| Q_3_ | |  | 0.65 (0.49-0.87) | 0.61 (0.52-0.73) | 0.75 (0.67-0.84) | 0.63 (0.44-0.90) |
| Q_4 (most optimistic)_ | |  | 0.60 (0.42-0.85) | 0.72 (0.59-0.88) | 0.71 (0.62-0.81) | 0.69 (0.46-1.03) |
| HR (95% CI) for an increase of one *SD* ^g^ | | | 0.81 (0.72-0.91) | 0.83 (0.78-0.88) | 0.85 (0.81-0.88) | 0.83 (0.73-0.94) |
| Q_1 (least optimistic)_ | **Model 3** | | 1 | 1 | 1 | 1 |
| Q_2_ |  |  | 0.75 (0.56-1.00) | 0.80 (0.68-0.94) | 0.81 (0.73-0.90) | 0.83 (0.58-1.18) |
| Q_3_ |  |  | 0.68 (0.51-0.91) | 0.65 (0.55-0.78) | 0.76 (0.68-0.85) | 0.63 (0.43-0.91) |
| Q_4 (most optimistic)_ |  |  | 0.63 (0.44-0.91) | 0.78 (0.64-0.96) | 0.72 (0.63-0.83) | 0.71 (0.46-1.07) |
| HR (95% CI) for an increase of one *SD* ^g^ | | | 0.83 (0.73-0.93) | 0.85 (0.80-0.91) | 0.85 (0.82-0.89) | 0.82 (0.72-0.94) |
| Risk estimates are Hazard ratios (HR) with 95% Confidence intervals (CI)  [a] self-reported physician/clinician diagnosis or a new regular use of antidepressants on biennial questionnaires or reporting clinical depressive symptoms according to the fifteen-item Geriatric Depression Scale (score≥6)  [b] 1,279 participants with missing information on birth region  [c] West= Oregon, Washington, California, Idaho, Montana, Wyoming, Colorado, Nevada, Utah, Arizona, New Mexico, Oklahoma, Texas, Alaska, Hawaii  [d] Midwest= Minnesota, Wisconsin, Indiana, Illinois, Michigan, Missouri, Indiana, Ohio, North Dakota, South Dakota, Nebraska, Kansas  [e] Northeast= New York, Pennsylvania, Maine, New Hampshire, Vermont, Massachusetts, Rhode Island, Connecticut, New Jersey  [f] South= Delaware, Maryland, Washington D.C., Virginia, West Virginia, North Carolina, South Carolina, Georgia, Florida, Kentucky, Tennessee, Alabama, Mississippi, Arkansas, Louisiana, Oklahoma  [g] of the standard (z-) score distribution of the optimism scale  ***Model.1:*** Age-adjusted [<65; 65-70; 71-75; 76-80, >80 years]  ***Model.2:*** Additionally adjusted for baseline depressive symptoms [continuous]  ***Model 3:*** *Model 2* + adjusted for educational status [Registered nurse; Bachelor´s degree; Advanced degree], race [Non-Hispanic white; black; other], subjective societal status [High; Medium-High; Medium-low or low], work status [Retired; Homemaker; Full/part time non nursing; Full/part time nursing], living arrangement [With spouse; Alone; Other], marital status [Married; Widowed; Other], husband´s educational status [High school graduate or less; College graduate; Graduate school] and father´s occupation [Professional or managerial; Clerical, sales or service; Other], bodily pain [None; Very mild/mild; Moderate; Severe/very severe], physical functioning [continuous], sleep duration [<7; 7-8; >8 hrs.], problem falling asleep or maintaining sleep [None of the time; A little of the time; Some/good bit of the time; Most/All of the time], providing care for grandchildren [None; Some; High] or an ill/disabled person [None; Some; High], multiple comorbidity [<2; ≥2 chronic diseases] and minor tranquilizers use [binary] | | | | | | |
|  | | | | | | |

| **Supplementary table 8**. Association of dispositional optimism and risk of incident depression ^a^ in the Nurses´ Health Study (*N*= 33,483) across race categories, follow-up 2004-2014. | | | | | |  |
| --- | --- | --- | --- | --- | --- | --- |
|  | |  | Race | | |  |
|  | |  | Non-Hispanic White  (*N*= 31,609) | Other  (*N*= 1,874) | |  |
| Cases/person-years | |  | 3840/273385 | 211/16006 | |  |
| Incident rate per 1000 person-years | | | 14.1 | 13.2 | |  |
| **Optimism quartiles** | |  |  |  |  | |
| First | | **Model 1:** age-adjusted | 1 | 1 | |  |
| Second | |  | 0.70 (0.65-0.76) | 0.66 (0.47-0.93) | |  |
| Third | |  | 0.56 (0.52-0.61) | 0.45 (0.31-0.65) | |  |
| Fourth | |  | 0.47 (0.43-0.52) | 0.36 (0.24-0.56) | |  |
| HR (95% CI) for an increase of one *SD* ^b^ | | | 0.74 (0.72-0.76) | 0.67 (0.59-0.75) | |  |
| First | | **Model 2:** Model 1 + baseline depressive symptoms | 1 | 1 | |  |
| Second | |  | 0.81 (0.74-0.88) | 0.79 (0.56-1.12) | |  |
| Third | |  | 0.71 (0.66-0.78) | 0.59 (0.66-0.78) | |  |
| Fourth | |  | 0.72 (0.65-0.80) | 0.55 (0.35-0.87) | |  |
| HR (95% CI) for an increase of one *SD* ^b^ | | | 0.85 (0.82-0.87) | 0.76 (0.66-0.86) | |  |
| First |  | | 1 | 1 | |  |
| Second |  | | 0.82 (0.75-0.89) | 0.76 (0.53-1.08) | |  |
| Third | **Model 3:** fully-adjusted | | 0.73 (0.67-0.80) | 0.67 (0.45-1.00) | |  |
| Fourth |  | | 0.74 (0.67-0.89) | 0.59 (0.53-1.08) | |  |
| HR (95% CI) for an increase of one *SD* ^b^ | | | 0.86 (0.83-0.88) | 0.79 (0.69-0.91) | |  |

Risk estimates are Hazard ratios (HR) with 95% Confidence intervals (CI).

[a] self-reported physician/clinician diagnosis or a new regular use of antidepressants on biennial questionnaires or reporting clinical depressive symptoms according to the fifteen-item Geriatric Depression Scale (score≥6)

[b] of the standard (z-) score distribution of the optimism scale

***Model.1:*** Age-adjusted [<65; 65-70; 71-75; 76-80, >80 years]

***Model.2:*** Additionally adjusted for baseline depressive symptoms [continuous]

***Model 3:*** *Model 2* + adjusted for educational status [Registered nurse; Bachelor´s degree; Advanced degree], region of birth [West; Midwest; Northeast; South], subjective societal status [High; Medium-High; Medium-low or low], work status [Retired; Homemaker; Full/part time non nursing; Full/part time nursing], living arrangement [With spouse; Alone; Other], marital status [Married; Widowed; Other], husband´s educational status [High school graduate or less; College graduate; Graduate school] and father´s occupation [Professional or managerial; Clerical, sales or service; Other], bodily pain [None; Very mild/mild; Moderate; Severe/very severe], physical functioning [continuous], sleep duration [<7; 7-8; >8 hrs.], problem falling asleep or maintaining sleep [None of the time; A little of the time; Some/good bit of the time; Most/All of the time], providing care for grandchildren [None; Some; High] or an ill/disabled person [None; Some; High], multiple comorbidity [<2; ≥2 chronic diseases] and minor tranquilizers use [binary]

| **Supplementary table 9**. Association of dispositional optimism and incident depression ^a^ risk in the Nurses´ Health Study (*N*=33,483), with a 2 year lag from baseline, 2006-2014. | | | | | | |
| --- | --- | --- | --- | --- | --- | --- |
|  | | Optimism quartiles | | | | Increase of one *standard deviation* ^b^ |
|  | | Q_1_  _(least optimistic)_  (*N* = 8,383) | Q_2_  (*N*= 7,941) | Q_3_  (*N*= 9,736) | Q_4_  _(most optimistic)_  (*N*= 7,423) |  |
| Cases/person-years | | 1319/53506 | 856/53637 | 803/67877 | 525/52407 |  |
| Incident rate per 1000 person-years | | 24.7 | 16.0 | 11.8 | 10.0 |  |
| **Model 1:** Age-adjusted model | HR (95% CI) | 1 | 0.67 (0.61-0.73) | 0.53 (0.48-0.57) | 0.45 (0.40-0.50) | 0.72 (0.70-0.75) |
| **Model 2:** Model1 + baseline depressive symptoms | HR (95% CI) | 1 | 0.77 (0.70-0.84) | 0.66 (0.61-0.73) | 0.68 (0.61-0.76) | 0.82 (0.79-0.85) |
| **Model 3:** Model2 + demographic covariates | HR (95% CI) | 1 | 0.77 (0.70-0.84) | 0.67 (0.61-0.73) | 0.69 (0.62-0.77) | 0.82 (0.79-0.85) |
| **Model 4:** Model2 + health depicting covariates | HR (95% CI) | 1 | 0.78 (0.71-0.85) | 0.68 (0.62-0.74) | 0.69 (0.62-0.77) | 0.83 (0.80-0.85) |
| **Model 5:** Fully-adjusted | HR (95% CI) | 1 | 0.77 (0.71-0.84) | 0.68 (0.62-0.74) | 0.70 (0.62-0.78) | 0.83 (0.80-0.85) |
| Risk estimates are Hazard ratios (HR) with 95% Confidence intervals (CI)  [a] self-reported physician/clinician diagnosis or a new regular use of antidepressants on biennial questionnaires or reporting clinical depressive symptoms according to the fifteen-item Geriatric Depression Scale (score≥6)  [b] of the standard (z-) score distribution of the optimism scale  ***Model.1:*** Age-adjusted [<65; 65-70; 71-75; 76-80, >80 years]  ***Model.2:*** Additionally adjusted for baseline depressive symptoms [continuous]  ***Model 3:*** *Model 2* + adjusted for educational status [Registered nurse; Bachelor´s degree; Advanced degree], region of birth [West; Midwest; Northeast; South], race [Non-Hispanic white; black; other], subjective societal status [High; Medium-High; Medium-low or low], work status [Retired; Homemaker; Full/part time non nursing; Full/part time nursing], living arrangement [With spouse; Alone; Other], marital status [Married; Widowed; Other], husband´s educational status [High school graduate or less; College graduate; Graduate school] and father´s occupation [Professional or managerial; Clerical, sales or service; Other]  ***Model 4:*** *Model 2* + adjusted for bodily pain [None; Very mild/mild; Moderate; Severe/very severe], physical functioning [continuous], sleep duration [<7; 7-8; >8 hrs.], problem falling asleep or maintaining sleep [None of the time; A little of the time; Some/good bit of the time; Most/All of the time], providing care for grandchildren [None; Some; High] or an ill/disabled person [None; Some; High], multiple comorbidity [<2; ≥2 chronic diseases] and minor tranquilizers use [binary]  ***Model 5:***  Includes all the covariates above | | | | | | |

| **Supplementary table 10**. Association of dispositional optimism and incident depression risk in the Nurse´s Health Study (*N*=33,483) ^a^, follow-up 2004-2014. The item “I felt hopeful about the future” was not included to calculate the CESD-10 score to adjust for baseline depressive symptoms since it might lead to/explain an overlap of the optimism and depression scores. | | | | | | |
| --- | --- | --- | --- | --- | --- | --- |
|  | | Optimism quartiles | | | | Increase of one *standard deviation* ^b^ |
|  | | Q_1_  _(least optimistic)_  (*N* = 8,383) | Q_2_    (*N*= 7,941) | Q_3_  (*N*= 9,736) | Q_4_  _(most optimistic)_  (*N*= 7,423) |  |
| Cases/person-years | | 1480/68783 | 1000/68131 | 958/86003 | 613/66474 |  |
| Incident rate per 1000 person-years | | 21.5 | 14.7 | 11.1 | 9.2 |  |
| **Model 1:** Age-adjusted model | HR (95% CI) | 1 | 0.70 (0.64-0.76) | 0.56 (0.51-0.60) | 0.46 (0.42-0.51) | 0.73 (0.72-0.76) |
| **Model 2:** Model1 + baseline depressive symptoms | HR (95% CI) | 1 | 0.77 (0.71-0.84) | 0.66 (0.61-0.72) | 0.65 (0.59-0.72) | 0.82 (0.79-0.84) |
| **Model 3:** Model2 + demographic covariates | HR (95% CI) | 1 | 0.77 (0.71-0.84) | 0.67 (0.62-0.73) | 0.66 (0.60-0.73) | 0.82 (0.79-0.84) |
| **Model 4:** Model2 + health depicting covariates | HR (95% CI) | 1 | 0.79 (0.73-0.86) | 0.69 (0.63-0.75) | 0.68 (0.61-0.75) | 0.83 (0.80-0.85) |
| **Model 5:** Fully-adjusted | HR (95% CI) | 1 | 0.79 (0.73-0.85) | 0.69 (0.63-0.75) | 0.68 (0.62-0.75) | 0.83 (0.80-0.85) |
| Risk estimates are Hazard ratios (HR) with 95% Confidence intervals (CI)  [a] sample size differs slightly because excluding the item affected exclusion criteria  [b] of the standard (z-) score distribution of the optimism scale  ***Model.1:*** Age-adjusted [<65; 65-70; 71-75; 76-80, >80 years]  ***Model.2:*** Additionally adjusted for baseline depressive symptoms [continuous]  ***Model 3:*** *Model 2* + adjusted for educational status [Registered nurse; Bachelor´s degree; Advanced degree], region of birth [West; Midwest; Northeast; South], race [Non-Hispanic white; black; other], subjective societal status [High; Medium-High; Medium-low or low], work status [Retired; Homemaker; Full/part time non nursing; Full/part time nursing], living arrangement [With spouse; Alone; Other], marital status [Married; Widowed; Other], husband´s educational status [High school graduate or less; College graduate; Graduate school] and father´s occupation [Professional or managerial; Clerical, sales or service; Other]  ***Model 4:*** *Model 2* + adjusted for bodily pain [None; Very mild/mild; Moderate; Severe/very severe], physical functioning [continuous], sleep duration [<7; 7-8; >8 hrs.], problem falling asleep or maintaining sleep [None of the time; A little of the time; Some/good bit of the time; Most/All of the time], providing care for grandchildren [None; Some; High] or an ill/disabled person [None; Some; High], multiple comorbidity [<2; ≥2 chronic diseases] and minor tranquilizers use [binary]  ***Model 5:***  Includes all the covariates above | | | | | | |

| **Supplementary table 11.** Association of dispositional optimism and incident depression ^a^ risk in the Nurse´s Health Study (*N*=33,483), follow-up 2004-2014. The item “Do you feel that your situation is hopeless?” was not included to calculate the Geriatric Depression Scale (GDS) score in 2008, 2012 and 2014 since it might represent optimism. | | | | | | |
| --- | --- | --- | --- | --- | --- | --- |
|  | | Optimism quartiles | | | | Increase of one *standard deviation* ^b^ |
|  | | Q_1_  _(least optimistic)_  (*N* = 8,383) | Q_2_    (*N*= 7,941) | Q_3_  (*N*= 9,736) | Q_4_  _(most optimistic)_  (*N*= 7,423) |  |
| Cases/person-years | | 1455/68849 | 992/68160 | 942/86042 | 611/66487 |  |
| Incident rate per 1000 person-years | | 21.1 | 14.6 | 11.0 | 9.2 |  |
| **Model 1:** Age-adjusted model | HR (95% CI) | 1 | 0.71 (0.65-0.76) | 0.56 (0.51-0.60) | 0.47 (0.43-0.52) | 0.74 (0.72-0.76) |
| **Model 2:** Model1 + baseline depressive symptoms | HR (95% CI) | 1 | 0.81 (0.75-0.88) | 0.71 (0.65-0.77) | 0.72 (0.65-0.79) | 0.84 (0.82-0.87) |
| **Model 3:** Model2 + demographic covariates | HR (95% CI) | 1 | 0.81 (0.75-0.88) | 0.71 (0.65-0.78) | 0.73 (0.66-0.81) | 0.85 (0.82-0.87) |
| **Model 4:** Model2 + health depicting covariates | HR (95% CI) | 1 | 0.82 (0.76-0.89) | 0.72 (0.67-0.79) | 0.74 (0.67-0.82) | 0.85 (0.83-0.88) |
| **Model 5:** Fully-adjusted | HR (95% CI) | 1 | 0.82 (0.76-0.89) | 0.73 (0.67-0.79) | 0.74 (0.67-0.82) | 0.85 (0.83-0.88) |
| Risk estimates are Hazard ratios (HR) with 95% Confidence intervals (CI)  [a] self-reported physician/clinician diagnosis or a new regular use of antidepressants on biennial questionnaires or reporting clinical depressive symptoms according to the fifteen-item Geriatric Depression Scale (score≥6)  [b] of the standard (z-) score distribution of the optimism scale  ***Model.1:*** Age-adjusted [<65; 65-70; 71-75; 76-80, >80 years]  ***Model.2:*** Additionally adjusted for baseline depressive symptoms [continuous]  ***Model 3:*** *Model 2* + adjusted for educational status [Registered nurse; Bachelor´s degree; Advanced degree], region of birth [West; Midwest; Northeast; South], race [Non-Hispanic white; black; other], subjective societal status [High; Medium-High; Medium-low or low], work status [Retired; Homemaker; Full/part time non nursing; Full/part time nursing], living arrangement [With spouse; Alone; Other], marital status [Married; Widowed; Other], husband´s educational status [High school graduate or less; College graduate; Graduate school] and father´s occupation [Professional or managerial; Clerical, sales or service; Other]  ***Model 4:*** *Model 2* + adjusted for bodily pain [None; Very mild/mild; Moderate; Severe/very severe], physical functioning [continuous], sleep duration [<7; 7-8; >8 hrs.], problem falling asleep or maintaining sleep [None of the time; A little of the time; Some/good bit of the time; Most/All of the time], providing care for grandchildren [None; Some; High] or an ill/disabled person [None; Some; High], multiple comorbidity [<2; ≥2 chronic diseases] and minor tranquilizers use [binary]  ***Model 5:***  Includes all the covariates above | | | | | | |

| **Supplementary table 12**. Association of optimism (score of the three positively worded items of the LOT-R), pessimism (score of the three negatively worded items of the LOT-R) and incident depression risk ^a^ in the Nurses´ Health Study (*N*=33,483), 2004-2014. | | | | | | |
| --- | --- | --- | --- | --- | --- | --- |
|  | | **Optimism quartiles (positively worded items of the LOT-R)** | | | | Increase of one *standard deviation* ^b^ |
|  | | Q_1_  **_(least optimistic)_**  (*N*=8,520) | Q_2_  (*N*=9,365) | Q_3_  (*N*=4,795) | Q_4_  **_(most optimistic)_**  (*N*=10,803) |  |
| Cases/person-years | | 1459/70469 | 1150/81156 | 492/41882 | 950/95879 |  |
| Incident rate per 1000 person-years | | 20.7 | 14.2 | 11.8 | 9.9 |  |
| **Model 1:** Age-adjusted model | HR (95% CI) | 1 | 0.70 (0.65-0.76) | 0.60 (0.54-0.66) | 0.50 (0.46-0.55) | 0.77 (0.75-0.79) |
| **Model 2:** Model1 + baseline depressive symptoms | HR (95% CI) | 1 | 0.79 (0.73-0.85) | 0.74 (0.66-0.82) | 0.72 (0.66-0.82) | 0.86 (0.84-0.89) |
| **Model 3:** Model2 + demographic covariates | HR (95% CI) | 1 | 0.79 (0.73-0.86) | 0.74 (0.67-0.82) | 0.73 (0.67-0.80) | 0.87 (0.84-0.89) |
| **Model 4:** Model2 + health depicting covariates | HR (95% CI) | 1 | 0.80 (0.74-0.86) | 0.75 (0.68-0.83) | 0.73 (0.67-0.79) | 0.87 (0.84-0.89) |
| **Model 5:** Fully-adjusted | HR (95% CI) | 1 | 0.80 (0.74-0.86) | 0.75 (0.68-0.83) | 0.73 (0.67-0.80) | 0.87 (0.84-0.90) |
|  |  | **Pessimism quartiles (negatively worded items of the LOT-R)** | | | |  |
|  |  | Q_1_  **_(most pessimistic)_**  (*N*=8,226) | Q_2_  (*N*=6,836) | Q_3_  (*N*=3,151) | Q_4_  **_(least pessimistic)_**  (*N*=15,270) | Increase of one *standard deviation* ^b^ |
| Cases/person-years | | 1369/68011 | 878/58411 | 355/27602 | 1449/135362 |  |
| Incident rate per 1000 person-years | | 20.1 | 15.0 | 12.9 | 10.7 |  |
| **Model 1:** Age-adjusted model | HR (95% CI) | 1 | 0.77 (0.71-0.84) | 0.68 (0.61-0.77) | 0.58 (0.54-0.62) | 0.80 (0.77-0.82) |
| **Model 2:** Model1 + baseline depressive symptoms | HR (95% CI) | 1 | 0.84 (0.77-0.92) | 0.77 (0.68-0.86) | 0.76 (0.71-0.82) | 0.89 (0.86-0.91) |
| **Model 3:** Model2 + demographic covariates | HR (95% CI) | 1 | 0.85 (0.78-0.92) | 0.77 (0.69-0.87) | 0.77 (0.72-0.84) | 0.89 (0.86-0.92) |
| **Model 4:** Model2 + health depicting covariates | HR (95% CI) | 1 | 0.85 (0.78-0.93) | 0.78 (0.70-0.88) | 0.79 (0.73-0.85) | 0.90 (0.87-0.92) |
| **Model 5:** Fully-adjusted | HR (95% CI) | 1 | 0.85 (0.78-0.93) | 0.79 (0.70-0.88) | 0.79 (0.73-0.86) | 0.90 (0.87-0.92) |
| CI=confidence interval; HR=hazard ratio.  [a] self-reported physician/clinician diagnosis or a new regular use of antidepressants on biennial questionnaires or reporting clinical depressive symptoms according to the fifteen-item Geriatric Depression Scale (score≥6)  [b] of the standard (z-) score distribution of the optimism (three positively worded LOT-R items)/pessimism (three negatively worded items LOT-R items) scale. Higher scores on the optimism scale indicate higher optimism while higher scores on the pessimism scale indicate less pessimism  ***Model.1:*** Age-adjusted [<65; 65-70; 71-75; 76-80, >80 years]  ***Model.2:*** Additionally adjusted for baseline depressive symptoms [continuous]  ***Model 3:*** *Model 2* + adjusted for educational status [Registered nurse; Bachelor´s degree; Advanced degree], region of birth [West; Midwest; Northeast; South], race [Non-Hispanic white; black; other], subjective societal status [High; Medium-High; Medium-low or low], work status [Retired; Homemaker; Full/part time non nursing; Full/part time nursing], living arrangement [With spouse; Alone; Other], marital status [Married; Widowed; Other], husband´s educational status [High school graduate or less; College graduate; Graduate school] and father´s occupation [Professional or managerial; Clerical, sales or service; Other]  ***Model 4:*** *Model 2* + adjusted for bodily pain [None; Very mild/mild; Moderate; Severe/very severe], physical functioning [continuous], sleep duration [<7; 7-8; >8 hrs.], problem falling asleep or maintaining sleep [None of the time; A little of the time; Some/good bit of the time; Most/All of the time], providing care for grandchildren [None; Some; High] or an ill/disabled person [None; Some; High], multiple comorbidity [<2; ≥2 chronic diseases] and minor tranquilizers use[binary]  ***Model 5:***  Includes all the covariates above | | | | | | |
